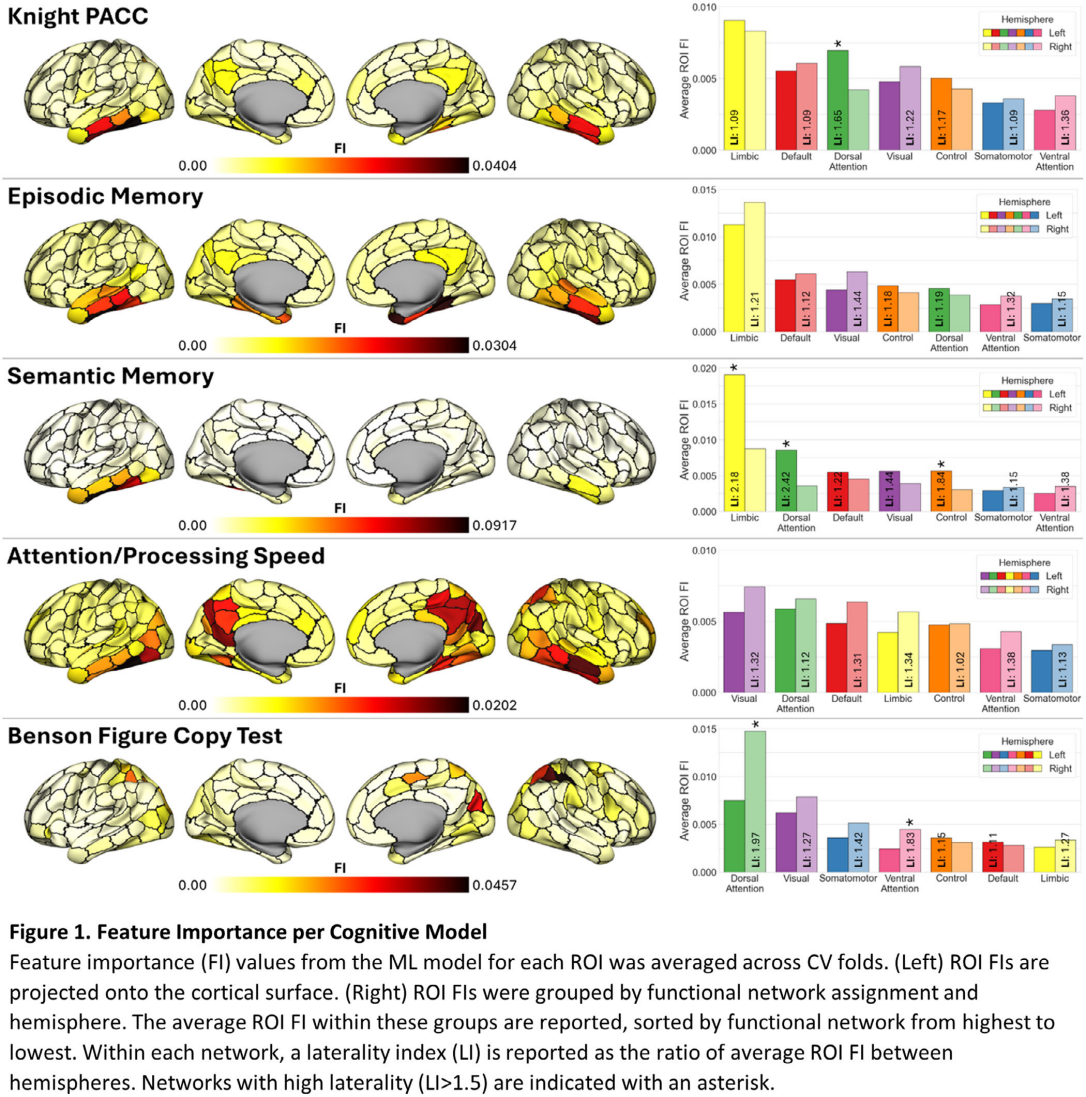# Tau spatial distribution predicts domain‐specific cognitive performance: a machine learning approach

**DOI:** 10.1002/alz70862_110262

**Published:** 2025-12-23

**Authors:** Stephanie Doering, Nicole S. McKay, Peter R Millar, Shaney Flores, Austin A. McCullough, Jason J. Hassenstab, John C. Morris, Brian A. Gordon, Tammie L.S. Benzinger

**Affiliations:** ^1^ Washington University in St. Louis, St. Louis, MO USA; ^2^ Washington University in St. Louis, School of Medicine, St. Louis, MO USA; ^3^ Department of Neurology, Washington University School of Medicine, St. Louis, MO USA; ^4^ Washington University School of Medicine, St. Louis, MO USA; ^5^ Washington University School of Medicine, Saint Louis, MO USA; ^6^ Washington University School of Medicine in St. Louis, St. Louis, MO USA

## Abstract

**Background:**

Patient cohorts exhibit heterogeneous regional tau spread correlating with specific clinical/cognitive profiles. We previously demonstrated tau spread was more sensitive to preclinical attention/processing speed deficits than tau burden, suggesting utility in patient‐specific evaluation and a wider scope of regions of interest (ROI) based on cognitive profile. Here, we implement a model framework to evaluate in which ROIs tau pathology best predicts impairment for various cognitive domains.

**Method:**

529 older adults were recruited with tau PET and neuropsychological testing from the Knight ADRC. Tau PET scans were parcellated using the Schaefer200 atlas. We implemented random forest regression within repeated k‐fold cross‐validation predicting cognitive score from ROI SUVRs for 5 cognitive measures: Knight Preclinical Alzheimer Cognitive composite (Knight PACC), Episodic Memory, Semantic Memory, Attention/Processing Speed, and Benson Figure Copy test (visuospatial). Model ROI feature importances (FI) were extracted using MDI. Regional patterns of FI were evaluated for each model based on functional network assignment, neuroanatomical location, and hemisphere lateralization.

**Result:**

FI spatial distributions differed by cognitive measure (Figure 1). For Knight PACC, high FI was found in several networks, the inferior temporal lobe, and bilateral. For Episodic Memory, high FI was found in the limbic network, the medial temporal lobe, and bilateral. For Semantic Memory, high FI was found in the limbic network, the inferior temporal lobe, and left‐lateralized. For Attention/Processing Speed, high FI was found in several networks, the medial parietal/lateral temporal lobes, and bilateral. For Benson Figure Copy, high FI was found in the dorsal attention network, the superior parietal lobe, and right‐lateralized.

**Conclusion:**

Different sets of brain regions best predict performance for each cognitive domain. Temporal lobe tau is highlighted for most domains, likely attributed to early accumulation. The parietal lobe is emphasized for attention and visuospatial measures, despite less tau. Semantic memory left‐lateralization may be attributed to language production. Visuospatial right‐lateralization likewise aligns with neuroimaging studies. Overall, the predictive regions of each cognitive measure align with functional connectivity studies, but constrained to regions with AD tau pathology. These results indicate high sensitivity of our cognitive measures to regional tau and the benefit of evaluating distinct brain regions depending on patient cognitive profiles.